# Genome-Wide Identification of CBL-CIPK Gene Family in Honeysuckle (*Lonicera japonica* Thunb.) and Their Regulated Expression Under Salt Stress

**DOI:** 10.3389/fgene.2021.751040

**Published:** 2021-11-02

**Authors:** Luyao Huang, Zhuangzhuang Li, Qingxia Fu, Conglian Liang, Zhenhua Liu, Qian Liu, Gaobin Pu, Jia Li

**Affiliations:** ^1^ School of Pharmacy, Shandong University of Traditional Chinese Medicine, Jinan, China; ^2^ School of Medicine and Pharmacy, Ocean University of China, Qingdao, China; ^3^ Department of Pharmacy, Linyi People’s Hospital, Linyi, China

**Keywords:** honeysuckle, calcineurin B-like protein, CBL-interacting protein kinase, salt stress, SOS pathway

## Abstract

In plants, calcineurin B-like proteins (CBLs) are a unique group of Ca^2+^ sensors that decode Ca^2+^ signals by activating a family of plant-specific protein kinases known as CBL-interacting protein kinases (CIPKs). CBL-CIPK gene families and their interacting complexes are involved in regulating plant responses to various environmental stimuli. To gain insight into the functional divergence of CBL-CIPK genes in honeysuckle, a total of six LjCBL and 17 LjCIPK genes were identified. The phylogenetic analysis along with the gene structure analysis divided both CBL and CBL-interacting protein kinase genes into four subgroups and validated by the distribution of conserved protein motifs. The 3-D structure prediction of proteins shown that most LjCBLs shared the same Protein Data Bank hit 1uhnA and most LjCIPKs shared the 6c9Da. Analysis of cis-acting elements and gene ontology implied that both LjCBL and LjCIPK genes could be involved in hormone signal responsiveness and stress adaptation. Protein-protein interaction prediction suggested that *LjCBL4* is hypothesized to interact with *LjCIPK7*/*9*/*15*/*16* and *SOS1*/*NHX1*. Gene expression analysis in response to salinity stress revealed that *LjCBL2*/*4*, *LjCIPK1*/*15*/*17* under all treatments gradually increased over time until peak expression at 72 h. These results demonstrated the conservation of salt overly sensitive pathway genes in honeysuckle and a model of Ca^2+^-*LjCBL4*/*LjSOS3*-*LjCIPK16*/*LjSOS2* module-mediated salt stress signaling in honeysuckle is proposed. This study provides insight into the characteristics of the CBL-CIPK gene families involved in honeysuckle salt stress responses, which could serve as a foundation for gene transformation technology, to obtain highly salt-tolerant medicinal plants in the context of the global reduction of cultivated land.

## Introduction

Calcium (Ca^2+^), whose level fluctuates when cells undergo external changes, acts as a universal secondary messenger for numerous signals and confers specific cellular responses (Ma L. et al., 2019). Ca^2+^ sensors or Ca^2+^-binding proteins can sense changes in cytoplasmic Ca^2+^ concentrations and transmit signals to regulate the function of the downstream proteins (such as transcriptional factors or membrane transporters) and elicit changes in cellular processes (such as gene expression or ionic fluxes) in response to environmental changes (Wang et al., 2020). In plants, there are three major classes of Ca^2+^-binding proteins include calmodulin (CaM) and CaM-like proteins (CMLs), calcineurin B-like proteins (CBLs), and calcium-dependent protein kinases (CDPKs) (Tang et al., 2020).

CBL proteins contain four typical helix-loop-helix motifs (EF-hands) for calcium-binding. However, unlike CDPKs, CBL proteins lack kinase activity and function in calcium signal transduction by interacting specifically with a group of Ser/Thr protein kinases, namely CBL-interacting protein kinases (CIPKs)/SNF1-related kinase group 3 (SnRK3s) (Hrabak et al., 2003). After sensing the change of Ca^2+^ level, CBLs physically interact with the NAF/FISL motifs at the C-terminal of CIPKs to activate CIPKs, and then, the activated CIPKs participate in calcium signaling by phosphorylating target proteins (Luan, 2009). Multiple experiments have proved the core role of the CBL-CIPK signal pathway in fine-tuning plants adaptive response to external environmental changes, including Arabidopsis, rice (Kolukisaoglu et al., 2004; Kanwar et al., 2014), maize (Chen et al., 2011), grapevine (Xi et al., 2017), turnip (Yin et al., 2017), eggplant (Li et al., 2016), and pineapple (Aslam et al., 2019). Among the 10 CBLs and 26 CIPKs in Arabidopsis, not only does each CBL interact with several CIPKs, but each CIPK interacts with one or more CBLs. Such interaction specificity and overlap between different members of CBL and CIPK family may confer both signaling specificity and functional synergism of CBL-CIPK complexes, forming a truly complex CBL-CIPK network when plants confronting a variety of external changes such as nutrient ions deprivation and abiotic stresses (Luan, 2009).

Soil salinization is one of the major environmental stress that reduces plant growth and productivity throughout the world, affecting an estimated 45 million hectares of irrigated land (Huang et al., 2019a). Under salt stress, extracellular high salt environment increased intracellular osmotic pressure and accumulated intracellular Na^+^ to a toxic level (Cheeseman, 1988; Deinlein et al., 2014). Although osmotic pressure and Na^+^ stress sensors in plants have not yet been identified, it seems that such stress signal transduction is closely related to Ca^2+^ pathway (Cheeseman, 1988). The SOS (salt overly sensitive) pathway was originally discovered from the model plant Arabidopsis and was a well-studied Ca^2+^-dependent CBL-CIPK module involved in salinity stress response regulation in plants (Olías et al., 2009). The SOS pathway, which involves two Ca^2+^ sensor proteins, *SOS3*/*AtCBL4* and *SCaBP8*/*AtCBL10*; the protein kinase *SOS2*/*AtCIPK24*; and the PM (plasma membrane)- Na^+^/H^+^ antiporter *SOS1*/*AtNHX7* (Liu et al., 2020). The Ca^2+^ signal induced by high-salt is decoded by *SOS3* and *SCaBP8*. *SOS3* recruit *SOS2* to the PM and activate its kinase activity. *SOS2*/*SOS3* complex then phosphorylates and activates downstream PM-binding *AtNHX7*/*SOS1* to transport Na^+^ out of the cell (Ji et al., 2013). Besides, *AtCIPK24* can interact with *AtCBL10*, then phosphorylates and activates the vacuolar NHX, pumping excess Na^+^ into the vacuole. The SOS pathway also exists in many other plants, such as rice, poplar (Tang et al., 2010), and spinach (Zhao et al., 2020). In rice, *OsCIPK24*/*OsSOS2* interacts with *OsCBL4*/*OsSOS3* regulating the activity of PM-located *OsSOS1*, thus participating in the regulation of plant salt tolerance (Pandey et al., 2015).

*Lonicera japonica* belongs to the honeysuckle family and is widely cultivated as an ornamental plant. Its dried flower buds have been prescribed in traditional Chinese medicine (TCM) to treat fever, influenza, sores and swelling for thousands of years (Pu et al., 2020). It is also a crucial antiviral drug used to treat the SARS coronavirus, influenza A viruses, the H1N1, H5N1, H7N9 flu virus, and Enterovirus 71 in recent years (Liu et al., 2005; Shang et al., 2011). It has further value as a component of cosmetic, healthy food, and beverages due to its unique aroma and pharmacological activity. Previously, Huang *et al.* found that the honeysuckle cultivar “Huajin 6” has high salt tolerance, but the physiological and molecular mechanisms are remained elusive until now (Huang et al., 2019b). Due to the great significance of CBL-CIPK networks in various physiological and stress resistance processes, it is of great significance to explore the salt tolerance mechanism of honeysuckle start from the analysis of CBL-CIPK gene.

In this study, we identified 6 CBL and 17 CIPK genes based on the genome sequences of honeysuckle. Various characteristics of these genes were analyzed, including physicochemical properties, phylogenetic relationship, conserved motifs, gene structures, cis-acting elements analysis, protein three-dimensional (3-D) structure prediction, and putative protein-protein interaction (PPI) prediction. Additionally, the gene expression patterns of CBL-CIPK genes were analyzed in different tissues and gradient salt stress. Our results could reveal the roles of CBL-CIPK genes in response to high-salt environments, and our study aimed to provide a valuable reference for further utilization of CBL-CIPK genes to develop salt-tolerant medicinal plants in the context of the global reduction of cultivated land.

## Materials and Methods

### Identification of CBL-CIPK Genes in Honeysuckle

The published CBL and CIPK gene sequences of *Arabidopsis* and *Oryza sativa* were downloaded from Arabidopsis Information Resource (TAIR) database (http://www.arabidopsis.org) and the rice genome database (http://rice.plantbiology.msu.edu//), respectively. The CBL and CIPK genes were further used as queries to search against *Lonicera japonica* genome databases to identify CBL-CIPK genes from honeysuckle. Then, The EF-hand calcium-binding domain (PS50222) of CBL proteins was determined based on PROSITE (https://prosite.expasy.org/) and HMMER (https://www.ebi.ac.uk/Tools/hmmer/). Each CIPK protein was subjected to the PROSITE (https://prosite.expasy.org/), NCBI Conserved Domain database (CDD, https://www.ncbi.nlm.nih.gov/Structure/cdd/wrpsb.cgi) and InterProscan (http://www.ebi.ac.uk/Tools/pfa/iprscan/) databases to confirm the presence of the pkinase domain (PF00069) and the NAF domain (PF03822). Finally, Expert Protein Analysis System (ExPASy, http://web.expasy.org/compute_pi/) was used to predicate the isoelectric point (pI) and molecular weight (M.W) (Bjellqvist et al., 1993; Bassil et al., 2019). The palmitoylation sites and myristoylation sites of CBLs were determined with GPS-Lipid 1.0 (http://lipid.biocuckoo.org/index.php) (Ren et al., 2008; Xie et al., 2016).

### Phylogenetic Analysis

The protein sequences of all the identified CBLs and CIPKs from *Lonicera japonica* (Lj), *Arabidopsis thaliana* (At), *Oryza sativa* (Os) were aligned using the Clustal-Omega (https://www.ebi.ac.uk/services). The whole phylogenetic trees were constructed by MEGA six using the Maximum likelihood (ML) method (Kumar et al., 2016). Pair distance among the CBL-CIPK genes was calculated by EMBOSS needle (https://www.ebi.ac.uk/Tools/psa/).

### Conserved Motifs, Gene Structures, and Cis-Acting Elements Analysis

Multiple Expectation Maximization for Motif Elicitation program (MEME version 5.0.5, http://meme-suite.org/tools/meme) was used to identify the conserved motifs of the CBL-CIPK proteins from honeysuckle (Bailey et al., 2009). The exon/intron organization for individual CBL-CIPK genes of honeysuckle were analyzed by the Gene Structure Display Serve (GSDS, http://gsds.cbi.pku.edu.cn/) (Hu et al., 2015). For cis-acting regulatory elements analysis, 2000 bp upstream of the coding sequence (CDS) was analyzed using PlantCARE (http://bioinformatics.psb.ugent.be/webtools/plantcare/html/) (Lescot et al., 2002).

### Chromosome Location

The chromosome location of CBL-CIPK genes were identified from the honeysuckle genome database. TBtools was used to draw the distribution graph of CBL-CIPK genes on chromosomes (Chen et al., 2020). MCScanX (http://chibba. pgml.uga.edu/mcscan2/) was used to analyze the gene duplication events (Wang et al., 2012). PAL2NAL program (http://www.bork.embl.de/pal2nal/) was used to calculate the rate of synonymous (Ks) and non-synonymous (Ka) substitution (Ks/Ka) (Goldman and Yang, 1994).

### Three-Dimensional Structural Prediction

The 3-D structure of CBL-CIPK proteins was modeled using the I-TASSER program (https://zhanglab.ccmb.med.umich.edu/I-TASSER/) (Yang et al., 2015).

### Protein-Protein Interaction Network Analysis

The PPI network of CBL-CIPK proteins was predicted using a model plant Arabidopsis on STRING protein interaction database (http://string-db.org) (Szklarczyk et al., 2019).

### Plant Material, Treatment, and qRT-PCR Analysis

The honeysuckle cultivar ‘Huajin 6’ was used in this study. For tissue-specific expression analysis, five tissues (flowering stage) including root, stem, mature leaf, young leaf, and flower were collected from 2-year-old honeysuckle grown at Shandong University of Traditional Chinese Medicine Medicinal Botanical Garden for quantitative real-time PCR (qRT-PCR). For salt stress, annual honeysuckle seedling plants were transplanted to plastic containers filled with quartz sand/vermiculite (1/3) in April 2021, one seedling per container. The seedlings were treated with the mixed solution containing NaCl (0, 100, 200, or 300 mM) and half-strength Hoagland’s nutrient solution. The seedlings were grown in climatic chambers (30°C day/25°C night, average RH of 65%, 300 μmol m^−2^· s^−1^ PAR, 14/10 h photoperiod). Samples for gene expression analysis were harvested at 0, 3, 6, 12, 24, 48 and 72 h after treatments and stored at –80°C until biochemical analysis. The roots were used for further study. Primers were designed using Primer Premier six and Oligo 7 with melting temperature of 58–62°C and production of 80–150 bp. The sequences of primers used in this study are listed in Supplementary Table S1 Sheet 2. 2^–ΔCt^ and 2^–ΔΔCt^ methods were applied to calculate the relative expression of genes in different tissues and gradient salt stress, respectively.

## Results

### Identification of LjCBLs and LjCIPKs Genes

A total of 6 CBL and 17 CIPK genes were finally obtained from the honeysuckle genome. All the LjCBLs contained the EF-hand while all the LjCIPKs possessed the conserved NAF/FISL motif at the C-terminal and the protein kinase domain at the N-terminal. These honeysuckle genes were named *LjCBL1*-*LjCBL6* and *LjCIPK1*-*LjCIPK17* respectively, according to their chromosomal positions.

As shown in Table 1, the deduced amino acid sequences of 6 LjCBL genes demonstrated great conservation in length, which ranged from 207 aa (*LjCBL5*) to 231 aa (*LjCBL2*), with an average length of 221 aa. The length of the LjCIPK genes was between 362 aa (*LjCIPK14*) and 519 aa (*LjCIPK3*), with an average length of 445 aa. The predicted M.W of the LjCBL proteins ranged from 23.82 to 26.62 kDa, and 41.06–58.35 kDa for CIPK proteins. The pI of the LjCBL proteins ranged from 4.64 to 5.43, with an average pI of 4.91, and 6.58 to 9.22 for CIPK proteins, with an average pI of 8.45. Overall, the PI of all LjCBL proteins was less than 7, while 88% of the LjCIPK proteins had a pI of greater than 7. Therefore, the LjCBL proteins are rich in acidic amino acids and the LjCIPKs are rich in basic amino acids. Additionally, *LjCBL1*/*2*/*4*/*6* have conserved myristoylation sites at their N-terminal, which play roles in protein-protein interactions and protein-membrane attachment; all LjCBLs are palmitoylated proteins.

**TABLE 1:** Characteristics of CBL and CIPK genes identified from honeysuckle.

(a) Characteristics of honeysuckle CBL genes										
Gene name	Gene ID	Chr	No. Amino acid	pI	Protein M.W (kDa)	Arabidopsis ortholog	Exon	EF-hands	Palmitoylation sites amino acid (location)	Myristoylation sites amino acid (location)
*LjCBL1*	Ljap00012651	1	225	4.68	25.74	At4g26570	8	4	C (4, 18)	G (7)
*LjCBL2*	Ljap00029480	1	231	5.43	26.62	At4g26570	8	4	C (5)	G (2)
*LjCBL3*	Ljap00022270	2	226	4.76	26.02	At5g55990	8	4	C (4, 12, 18)	\
*LjCBL4*	Ljap00010024	4	221	4.64	25.52	At5g24270	8	4	C (3, 5)	G (2)
*LjCBL5*	Ljap00010536	7	207	5.23	23.82	At4g33000	8	4	C (16, 36, 39)	\
*LjCBL6*	Ljap00011794	9	213	4.74	24.26	At5g47100	8	4	C (3)	G (2)

(b) Characteristics of honeysuckle CIPK genes									
Gene name	Gene ID	Chr	No. Amino acid	pI	Protein M.W (kDa)	Arabidopsis ortholog	Exon	Protein kinase domain	NAF motif
*LjCIPK1*	Ljap00011922	1	430	6.58	48.96	At2g30360	2	24–278	305–361
*LjCIPK2*	Ljap00011917	1	455	9.10	51.82	At5g58380	1	12–266	313–368
*LjCIPK3*	Ljap00029001	1	519	8.80	58.35	At5g58380	2	60–314	361–409
*LjCIPK4*	Ljap00029003	1	429	8.08	48.77	At5g01820	1	23–277	302–358
*LjCIPK5*	Ljap00011214	2	434	9.05	48.27	At2g30360	1	30–284	311–367
*LjCIPK6*	Ljap00011209	2	448	9.22	51.00	At5g58380	1	12–266	311–366
*LjCIPK7*	Ljap00031638	2	436	8.57	49.27	At5g58380	1	12–266	306–360
*LjCIPK8*	Ljap00019729	2	474	8.75	53.83	At4g18700	2	31–285	342–396
*LjCIPK9*	Ljap00009548	3	438	8.44	49.67	At5g01820	1	30–284	313–366
*LjCIPK10*	Ljap00017728	4	432	9.18	48.43	At4g30960	2	14–268	300–358
*LjCIPK11*	Ljap00014653	4	442	8.60	50.35	At5g10930	2	25–280	312–366
*LjCIPK12*	Ljap00030195	5	477	6.88	54.62	At4g24400	14	9–278	324–380
*LjCIPK13*	Ljap00029708	7	446	8.57	50.62	At5g10930	1	11–266	297–352
*LjCIPK14*	Ljap00016707	7	362	7.05	41.06	At1g01140	13	27–234	274–332
*LjCIPK15*	Ljap00019130	7	428	9.19	48.21	At4g30960	3	15–269	299–357
*LjCIPK16*	Ljap00025477	7	490	9.07	55.67	At5g35410	13	11–292	338–394
*LjCIPK17*	Ljap00033052	8	423	8.57	47.04	At3g23000	2	23–278	320–358

### Phylogenetic Analysis

**FIGURE 1:**
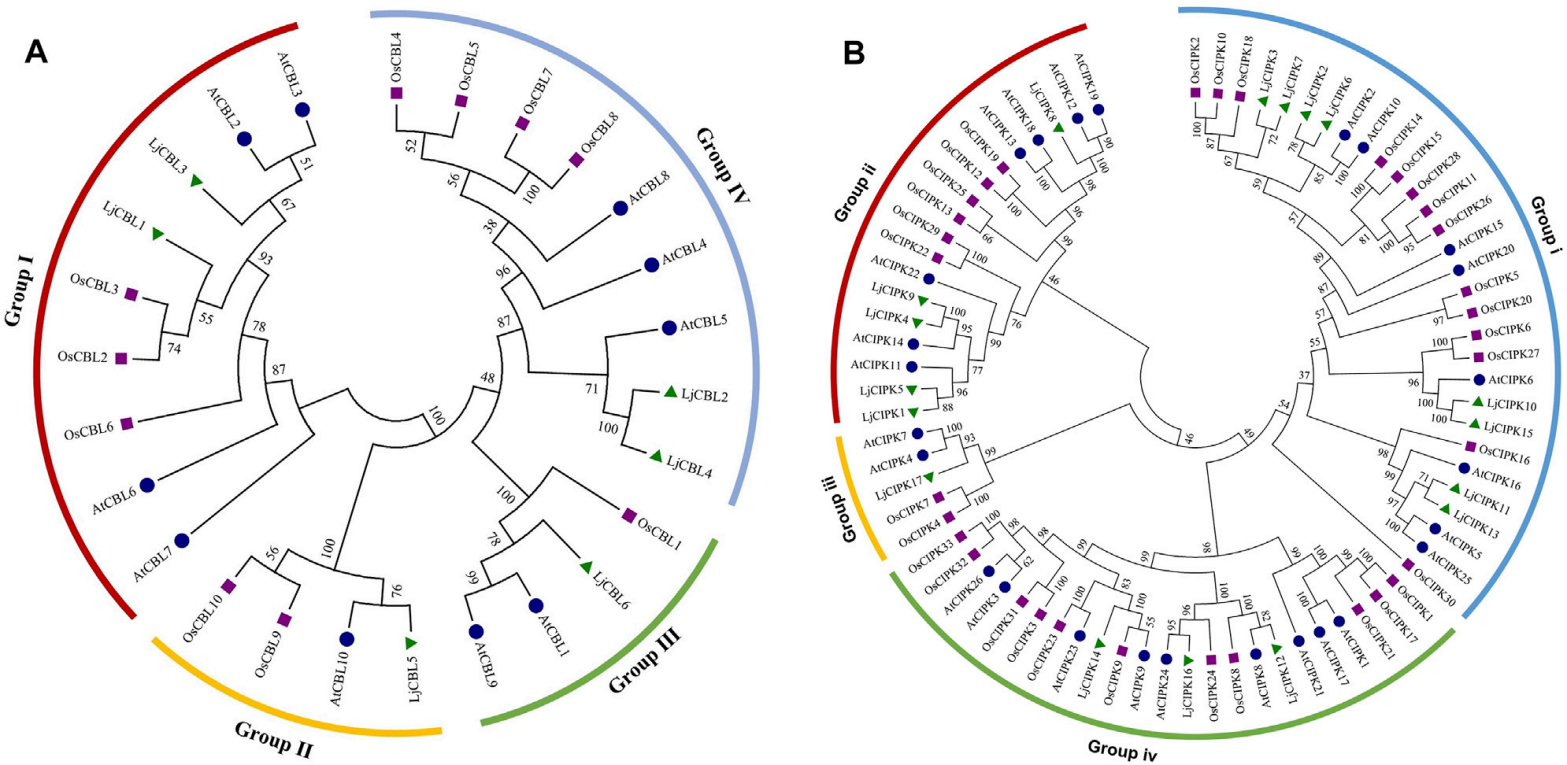
Phylogenetic tree of the CBL **(A)** proteins and CIPK **(B)** proteins from *Lonicera japonica* (Lj), *Arabidopsis thaliana* (At), *Oryza sativa* (Os). The different colored arcs indicate different subgroups. Proteins from honeysuckle, Arabidopsis, and rice are denoted by green triangles, blue circles, and purple squares, respectively. Details of CBL and CIPK protein sequence from three species are listed in Supplementary Table S1 Sheet 1.

To determine evolutionary relationships and functional associations, multi-species phylogenetic trees using the identified CBL and CIPK full-length protein sequences from *Lonicera japonica* (Lj), *Arabidopsis thaliana* (At), *Oryza sativa* (Os) were constructed (Figure 1). Multiple protein sequence alignment showed that both CBLs and CIPKs were clustered into four subgroups. There are two LjCBL (*LjCBL1*/*3*) proteins in Group I, one LjCBL (*LjCBL5*) protein in group II, one LjCBL (*LjCBL6*) protein in group III, and two LjCBL (*LjCBL2*/*4*) proteins in group IV. Among CIPK subgroups, the Group i had the largest number of members, with eight LjCIPK (*LjCIPK2*/*3*/*6*/*7*/*10*/*11*/*13*/*15*) proteins. Group iii had the fewest members, with only one LjCIPK (*LjCIPK17*) protein. Group ii and Group iv contained five LjCIPK (*LjCIPK1*/*4*/*5*/*8*/*9*) and three LjCIPK (*LjCIPK12*/*14*/*16*) proteins respectively.

The conservation of sequence among the CBL-CIPK genes was also confirmed by the identities and similarities of amino acid sequences (Supplementary Table S1 Sheet 3 and Sheet 4). The results showed that the identity of different LjCBLs ranged from 29.6 to 89.4% and the identity of different LjCIPKs ranged from 33.6 to 82.7%. The LjCBLs clustered into the same subgroup display higher identities of sequence in amino acid level (*LjCBL1*/*LjCBL3* = 89.4% and *LjCBL2*/*LjCBL4* = 82.2%), whilst the LjCBL genes in different subgroups exhibit lower identities. The sequences of *LjCIPK10*/*LjCIPK15* and *LjCIPK4*/*LjCIPK9* have higher identities (82.7 and 72.1%), which were the members of a close evolutionary relationship.

### Chromosomal Location, Ka/Ks Ratio Calculation

**FIGURE 2:**
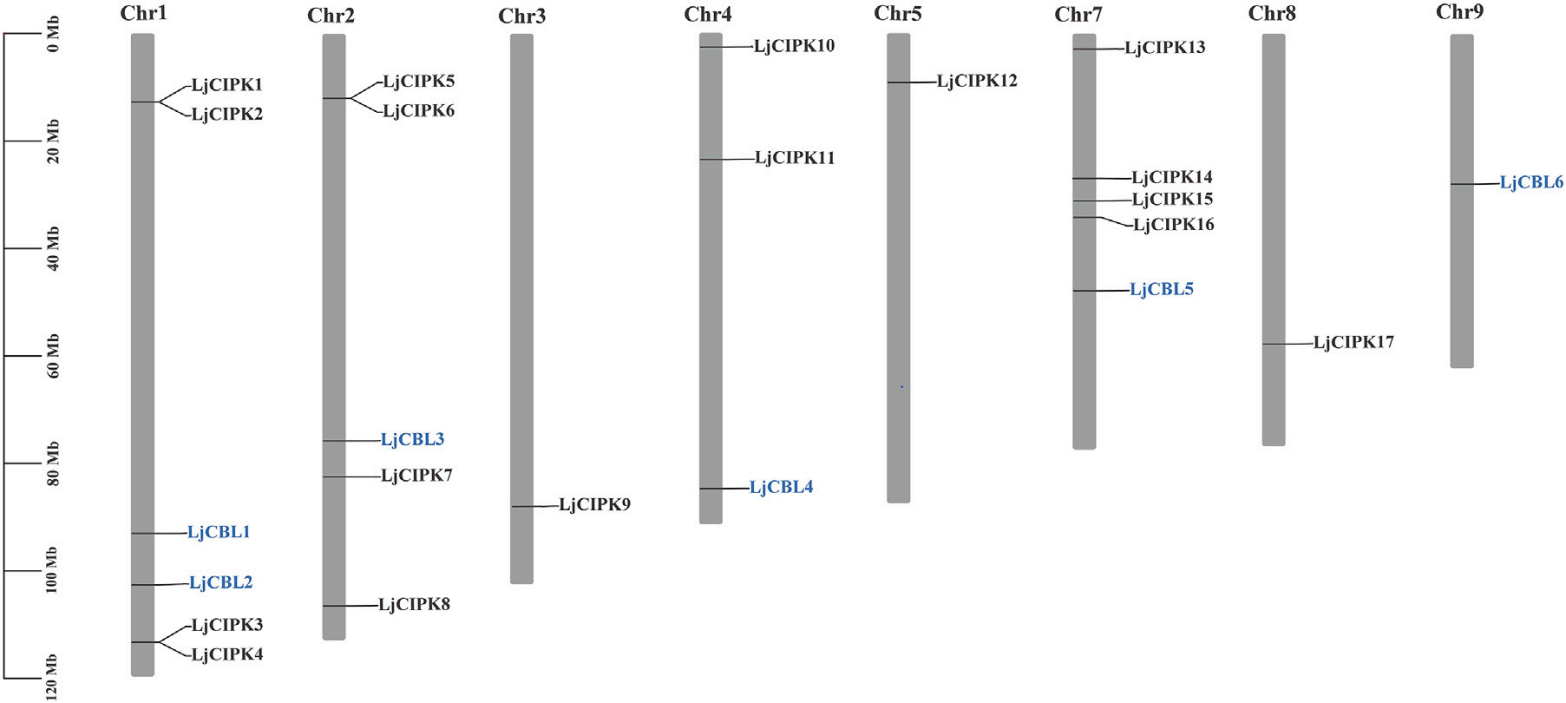
Physical mapping of honeysuckle CBL-CIPK genes. The numbers of CBL-CIPK genes are indicated on the right of each chromosome. The scale on the left represents chromosome length.

As shown in Figure 2, six LjCBL genes were mapped onto five of total nine honeysuckle chromosomes while seventeen LjCIPK genes were mapped onto eight of total nine chromosomes, indicating a diverse distribution. *LjCIPK1* and *LjCIPK2* on chromosome 1 overlapped, but the two genes are located far away in the phylogenetic tree, indicating that their biological functions may be different. The same overlap was also observed on chromosome 1 (*LjCIPK3* and *LjCIPK4*) and chromosome 2 (*LjCIPK5* and *LjCIPK6*). In the present study, only one (*LjCIPK10*/*LjCIPK15*) duplicated gene pair was identified (Supplementary Table S1 Sheet 5). Ka/Ks ratio between *LjCIPK10* and *LjCIPK15* is 0.0561, implying that the genes had undergone strong purifying selection pressure, which reduces the rate of change in amino acid profile.

### Gene Structure and Conserved Motifs Analysis

**FIGURE 3:**
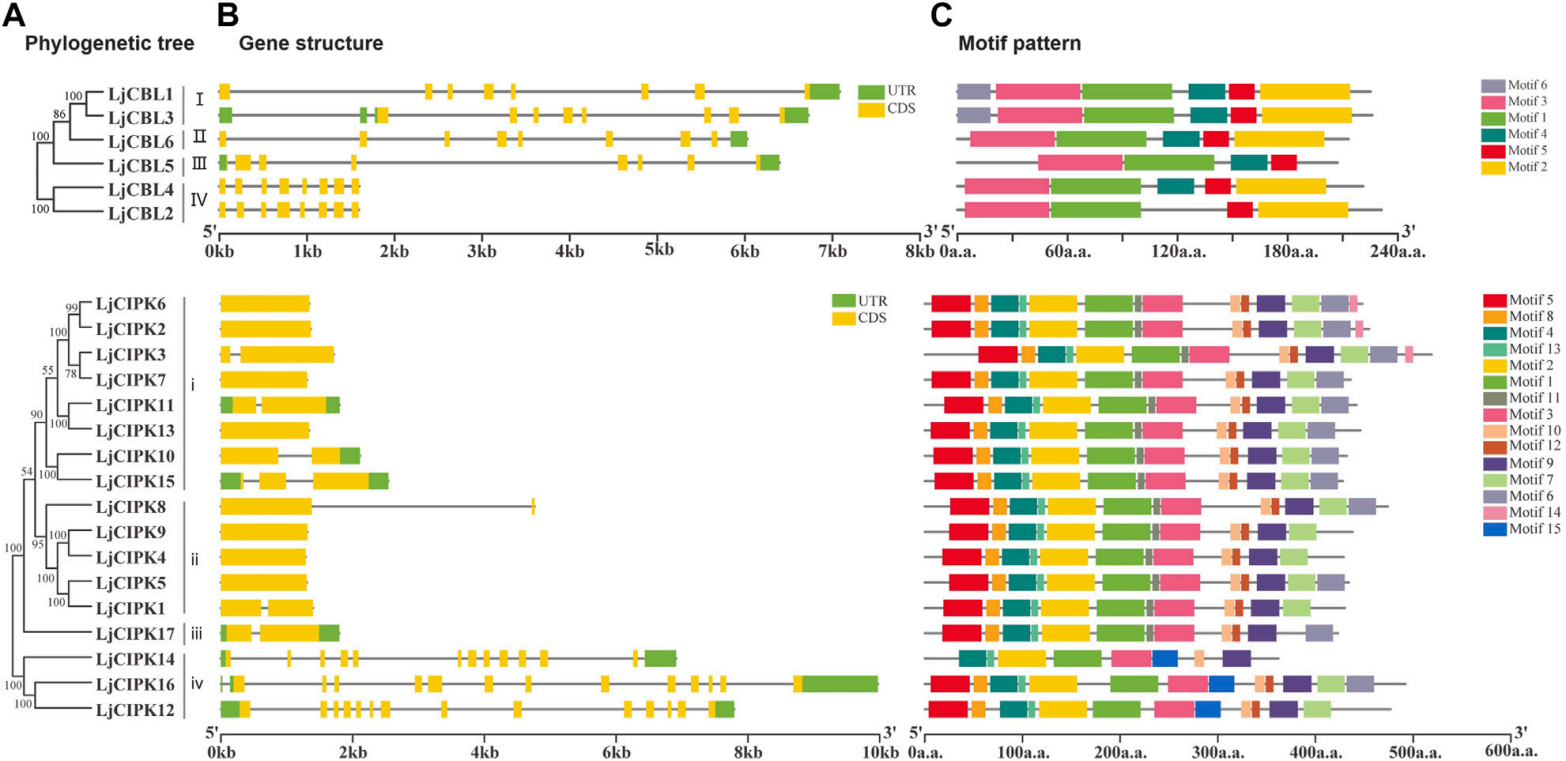
Phylogenetic relationships(A), gene structure(B), and conserved motifs(C) in CBL-CIPK genes from honeysuckle **(A)** Phylogenetic relationships **(B)** gene structure. Green boxes indicate untranslated 5′- and 3′-regions, yellow boxes indicate exons, and black lines indicate introns. The length of the protein can be estimated using the scale at the bottom **(C)** Conserved motifs. The motifs are displayed in different colored boxes. The sequence information for each motif is provided in Supplementary Table S1, Sheet 6 and Supplementary Figures S1,S2.

Members with close evolutionary relationships shown uniform or similar gene structure and motif composition (Figure 3B). The gene structure analysis along with the phylogeny results showed that the genes with a similar intron/exon pattern clustered near to each other in the same groups. LjCBL genes contained 8 exons and seven to nine introns. Group i/ii/iii of LjCIPK genes possessed one to three exons and one to two introns, whilst Group iv of LjCIPK genes had 13–14 exons and 12–13 introns. In general, the exon length, exons/intron number were moderately conserved among the various subgroups, indicating similar biological function.

To further investigate the characteristic region of CBL-CIPK proteins, the motif distributions were investigated (Figure 3C). A total of six putative motifs were identified in LjCBL proteins, and 15 motifs were identified in LjCIPK proteins. In the LjCBL family, three motifs (motif 1,3, and 5) existed in all the members, whilst motif 6 was only detected in Group I. Motif 2 existed in all subgroups except group III. In the LjCIPK family, seven motifs (motif 1, 2, 3,4, 9,10, and 13) existed in all the members, including the NAF motif (motif 10) and Protein kinase domain (motif 1, 2). Motif 11 was existed in both Group i and ii, while motif 15 was only detected in Group iv, suggesting that they might perform group-specific functions. The results indicate that members of the CBL-CIPK family belonging to the same subgroup have very similar motif types and numbers, but there are also differences in motif patterns among members of the same subgroup.

### Cis-Acting Elements Analysis

To further investigate the potential regulatory mechanism of the CBL-CIPK genes in honeysuckle, the cis-acting regulatory elements of the 2000 bp upstream region from the translation initiation site of the CBL-CIPK genes were surveyed (Table 2). In the CBL-CIPK promoters, seven hormone-related (e.g., ABRE, TCA-element, CGTCA-motif), seven stress-related (e.g., LTR, ARE, WUN-motif, MBS), and twelve development-related (e.g., G-box, GT1-motif) elements were identified. There were half of the LjCBL genes that contained more than five types of hormone-related cis-acting elements, but only *LjCIPK9* in the LjCIPK family contained so many types. Among stress-related cis-acting elements, ARE and STRE were found in most LjCBL and LjCIPK promoters. *LjCBL4* contained nine stress-response elements, including four ARE, one WUN-motif, and four STRE. *LjCIPK13* contained fourteen stress-response elements, including five STRE, four ARE, two WUN-motif, 2 W box, and one TC-rich repeats, which was the largest number of stress-response elements contained within one gene. *LjCIPK6*/*8*/*16* contained ten stress-response elements respectively, which is slightly less than *LjCIPK13*. Among development-related cis-acting elements, the circadian, TCT-motif, and GCN4 motif were only predicted in CIPK genes, but not found in CBL genes. These results implied that both LjCBL and LjCIPK genes could be involved in hormone signal responsiveness and stress adaptation.

**TABLE 2:** Kinds and amounts of hormone-, stress-, and development-related cis-acting element in the promoters of CBL-CIPK genes of honeysuckle.

Functional class	Elements	Function	LjCBLs						LjCIPKs																
			1	2	3	4	5	6	1	2	3	4	5	6	7	8	9	10	11	12	13	14	15	16	17
Hormone	ABRE	ABA-responsive ele`ment	0	1	1	0	3	1	3	0	2	0	2	1	0	3	4	2	1	2	4	5	10	0	2
	TCA-element	salicylic acid-responsive element	0	2	1	0	1	2	0	1	0	1	2	3	1	1	1	1	0	1	0	0	0	2	0
	P-box	gibberellin-responsive element	0	1	1	0	0	0	0	0	0	0	0	0	0	1	0	0	1	0	0	0	0	1	0
	TATC-box	gibberellin-responsive element	5	1	0	0	0	1	1	0	0	0	0	0	0	0	1	2	0	0	1	0	0	0	1
	CGTCA-motif	MeJA-responsive element	2	2	1	0	3	1	1	0	3	0	2	1	0	0	2	0	0	0	1	1	0	0	1
	TGACG-motif	MeJA-responsive element	2	2	1	0	3	1	1	0	3	0	2	1	0	0	2	0	0	0	1	1	0	0	1
	TGA-element	auxin-responsive element	1	0	0	1	0	0	0	0	0	0	0	0	0	0	0	0	1	1	0	1	1	0	0
Stress	ARE	anaerobic induction	0	2	0	4	3	3	1	0	3	3	2	1	1	4	1	0	1	0	4	1	2	4	1
	LTR	low-temperature responsiveness	0	0	2	0	1	0	0	0	1	0	0	3	0	0	0	1	0	0	0	2	1	1	1
	MBS	MYB binding site involved in drought-inducibility	1	1	1	0	0	0	0	0	0	1	0	0	2	0	0	0	1	0	0	0	0	1	1
	TC-rich repeats	defense and stress responsive element	0	0	0	0	1	2	0	1	0	0	0	3	0	0	0	1	0	0	1	0	0	0	1
	W box	WRKY Transcription factor binding site	1	0	2	0	0	0	2	0	0	0	1	2	0	0	1	0	2	0	2	1	1	2	0
	WUN-motif	wound-responsive element	0	2	0	1	1	0	1	1	0	1	0	0	0	1	1	0	0	0	2	0	2	0	0
	STRE	Stress response element	1	1	0	4	0	2	0	0	2	3	4	1	2	5	0	1	5	3	5	3	3	2	4
Others	MYB	Transcription factor	4	5	3	7	3	3	4	6	2	3	7	7	8	3	4	6	4	3	3	1	4	2	2
	AE-box	light-responsive element	1	1	1	0	1	1	0	0	0	0	0	2	0	0	1	0	0	1	0	0	0	0	0
	GATA-motif	light-responsive element	3	0	2	0	1	0	0	0	0	1	0	1	0	0	2	0	0	1	0	1	0	1	0
	G-box	light-responsive element	1	1	1	0	3	0	1	0	2	0	3	1	1	6	4	3	1	1	5	4	13	0	3
	GT1-motif	light-responsive element	2	0	1	2	0	1	2	1	1	1	2	0	0	3	0	1	0	0	2	2	0	2	1
	TCT-motif	light-responsive element	—						2	1	1	1	1	2	1	1	0	0	1	2	1	0	1	0	3
	Box 4	light responsiveness	4	2	3	2	11	0	7	1	2	1	2	3	2	0	4	6	2	3	2	0	3	3	4
	MRE	MYB binding site involved in light responsiveness	0	1	0	1	0	0	0	1	1	0	0	0	0	1	0	0	1	0	1	0	0	3	1
	circadian	circadian control	—						0	1	0	0	0	1	0	0	0	0	1	0	0	0	0	0	0
	CAT-box	meristem expression	0	1	0	0	0	1	0	0	0	1	0	0	1	0	0	0	0	0	0	0	0	0	1
	GCN4_motif	endosperm expression	—						0	2	0	0	0	0	0	0	0	0	0	0	0	0	0	0	0
	O2-site	zein metabolism regulation	0	0	0	0	0	1	0	0	1	1	0	1	1	0	3	0	1	0	0	0	0	0	0

### Proteins Structures Analysis

**FIGURE 4:**
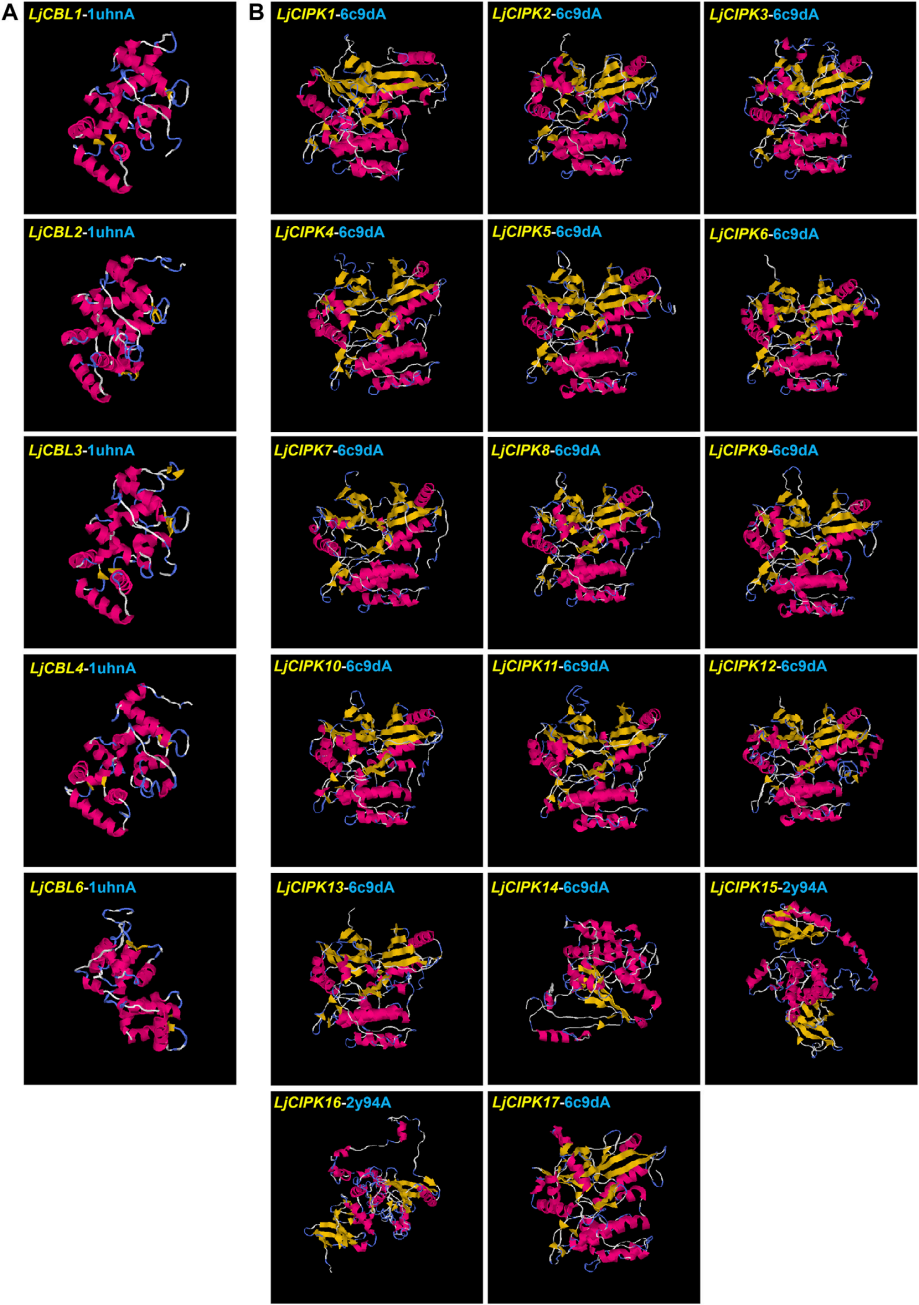
Structural analysis of six LjCBL **(A)** and seventeen LjCIPK **(B)** modeled proteins. The α-helix, β-strand, and random coil are marked by red, yellow and blue, respectively. The parameters of the best PDB structure for LjCBLs and LjCIPKs are listed in Table 3. Details of secondary structure are shown in Supplementary Figures S3,S4.

To gain more insight into the putatively functional mechanism of CBL-CIPK proteins in honeysuckle, all the proteins were modeled by I-TASSER (Figure 4). The 3-D structures were construed based on the best structural templates and crystal structures from Protein Data Bank (PDB). The parameters of the best PDB structure (Table 3) illustrated that the models were constructed with high credibility since all of them had a C-score varied from −2.63 to 0.26. Most LjCBL proteins shared the same PDB hit 1uhnA (The crystal structure of the calcium-binding protein *AtCBL2* from *Arabidopsis thaliana*) and LjCIPK proteins shared the 6c9Da (Crystal structure of KA1-autoinhibited MARK1 kinase). The same PDB hit indicating that their 3-D structures were similar.

**TABLE 3:** Structural dependent modeling parameters for the CBL-CIPK proteins.

Protein	C-score	TM-score	RMSD (Å)	Best identified structural analogs in PDB				
				PDB hit	TM-score	RMSD	IDEN	Cov
*LjCBL1*	−0.68	0.63 ± 0.14	7.0 ± 4.1Å	1uhnA	0.838	0.27	0.931	0.84
*LjCBL2*	−0.80	0.61 ± 0.14	7.4 ± 4.2Å	1uhnA	0.809	0.61	0.55	0.818
*LjCBL3*	−0.92	0.60 ± 0.14	7.6 ± 4.3Å	1uhnA	0.835	0.26	0.968	0.836
*LjCBL4*	−0.62	0.63 ± 0.13	6.9 ± 4.1Å	1uhnA	0.852	0.35	0.582	0.855
*LjCBL5*	−2.63	0.41 ± 0.14	11.5 ± 4.5Å	1v1gA	0.664	2.43	0.553	0.768
*LjCBL6*	−0.49	0.65 ± 0.13	6.5 ± 3.9Å	1uhnA	0.885	0.26	0.698	0.887
*LjCIPK1*	−0.43	0.66 ± 0.13	7.9 ± 4.4Å	6c9dA	0.890	2.14	0.326	0.928
*LjCIPK2*	−0.87	0.60 ± 0.14	9.1 ± 4.6Å	6c9dA	0.920	0.98	0.270	0.927
*LjCIPK3*	−2.10	0.46 ± 0.15	12.5 ± 4.3Å	6c9dA	0.803	0.780	0.305	0.807
*LjCIPK4*	−0.14	0.70 ± 0.12	7.3 ± 4.2Å	6c9dA	0.906	1.140	0.322	0.921
*LjCIPK5*	0.110	0.73 ± 0.11	6.8 ± 4.0Å	6c9dA	0.906	1.080	0.304	0.917
*LjCIPK6*	−0.290	0.68 ± 0.12	7.7 ± 4.3Å	6c9dA	0.926	0.750	0.286	0.933
*LjCIPK7*	0.030	0.72 ± 0.11	6.9 ± 4.1Å	6c9dA	0.933	1.100	0.295	0.947
*LjCIPK8*	−0.55	0.64 ± 0.13	8.4 ± 4.5Å	6c9dA	0.878	0.990	0.295	0.888
*LjCIPK9*	−0.26	0.68 ± 0.12	7.6 ± 4.3Å	6c9dA	0.887	1.240	0.322	0.902
*LjCIPK10*	0.26	0.75 ± 0.10	6.4 ± 3.9Å	6c9dA	0.928	1.12	0.292	0.944
*LjCIPK11*	−0.440	0.66 ± 0.13	8.0 ± 4.4Å	6c9dA	0.906	1.160	0.305	0.919
*LjCIPK12*	−1.250	0.56 ± 0.15	10.1 ± 4.6Å	6c9dA	0.862	1.140	0.301	0.876
*LjCIPK13*	−0.710	0.62 ± 0.14	8.7 ± 4.5Å	6c9dA	0.902	0.990	0.290	0.913
*LjCIPK14*	−0.670	0.63 ± 0.14	8.1 ± 4.4Å	6c9dA	0.929	1.600	0.285	0.970
*LjCIPK15*	−1.240	0.56 ± 0.15	9.8 ± 4.6Å	2y94A	0.826	2.750	0.318	0.899
*LjCIPK16*	−1.880	0.49 ± 0.15	11.8 ± 4.5Å	2y94A	0.816	1.080	0.325	0.829
*LjCIPK17*	−1.740	0.50 ± 0.15	11.0 ± 4.6Å	6c9dA	0.891	1.150	0.308	0.903

### Protein-Protein Interaction Prediction

**FIGURE 5:**
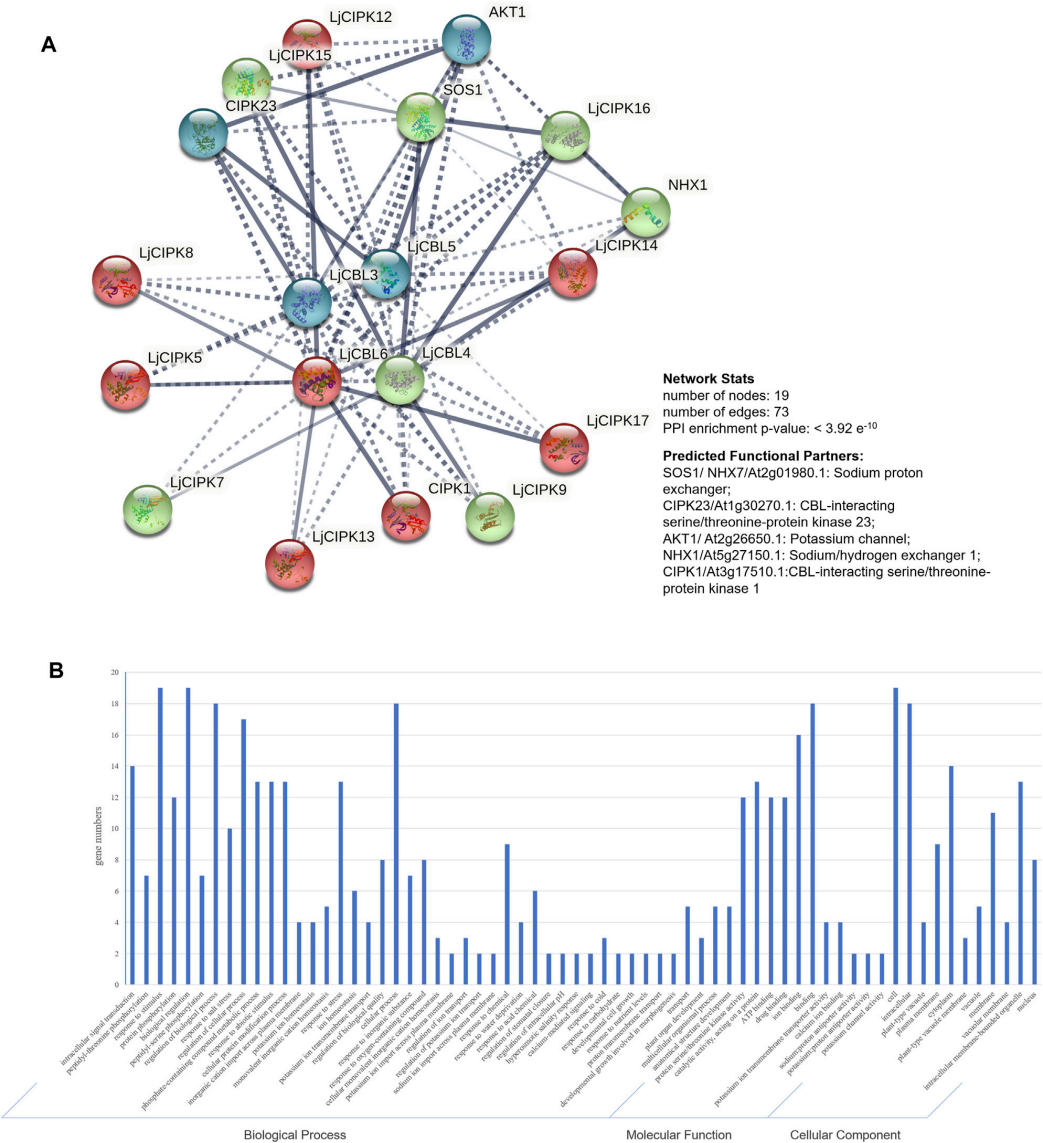
Protein-protein interaction (PPI) prediction of LjCBLs and LjCIPKs **(A)** PPI network. Line thickness indicates the strength of data support. The network is clustered into 3 clusters, which are represented with red, green, and blue nodes, respectively **(B)** Gene Ontology (GO) analysis of the genes from the PPI network.

To further explore the potential function of LjNHX members, the PPI network was constructed with STRING database, which was based on either known experimental or predicted interactions. Because the PPI network of *Lonicera japonica* is not available in the STRING database so far, we used the homolog gene between *Arabidopsis thaliana* and *Lonicera japonica* to search in the database. As shown in Figure 5, no immediately interacted relationship was predicted among LjCBLs or LjCIPKs. However, LjCBL and LjCIPK proteins have significantly more interactions. Individual *LjCBL6* protein was hypothesized to interact with *LjCIPK5*/*8*/*12*/*13*/*14*/*17* and *CIPK1*, and these proteins were clustered to red cluster in the PPI network. *LjCBL4* protein was hypothesized to interact with *LjCIPK7*/*9*/*15*/*16* and *SOS1*/*NHX1*, and these proteins were clustered to green cluster. However, individual *LjCBL5* only interacts with *CIPK23* and *AKT1*, and *LjCBL3* was hypothesized to interact with *CIPK23* and *AKT1*, these proteins were clustered into the blue cluster.

Then, genes from PPI network were described using three categories of GO classification: molecular function (MF), biological processes (BP) and cellular components (CC). The results of GO analysis showed that the proteins involved in the network were mainly localized to the cytoplasm, membrane, nucleus, and intracellular membrane-bounded organelle. Regarding MF, most of the proteins possessed protein serine/threonine kinase activity, catalytic activity, ion binding, and ion transmembrane transporter activity. Regarding BP, most of the proteins mainly participate in intracellular signal transduction, plant development process and respond to external stimuli.

### Expression Patterns of LjCBLs and LjCIPKs

**FIGURE 6:**
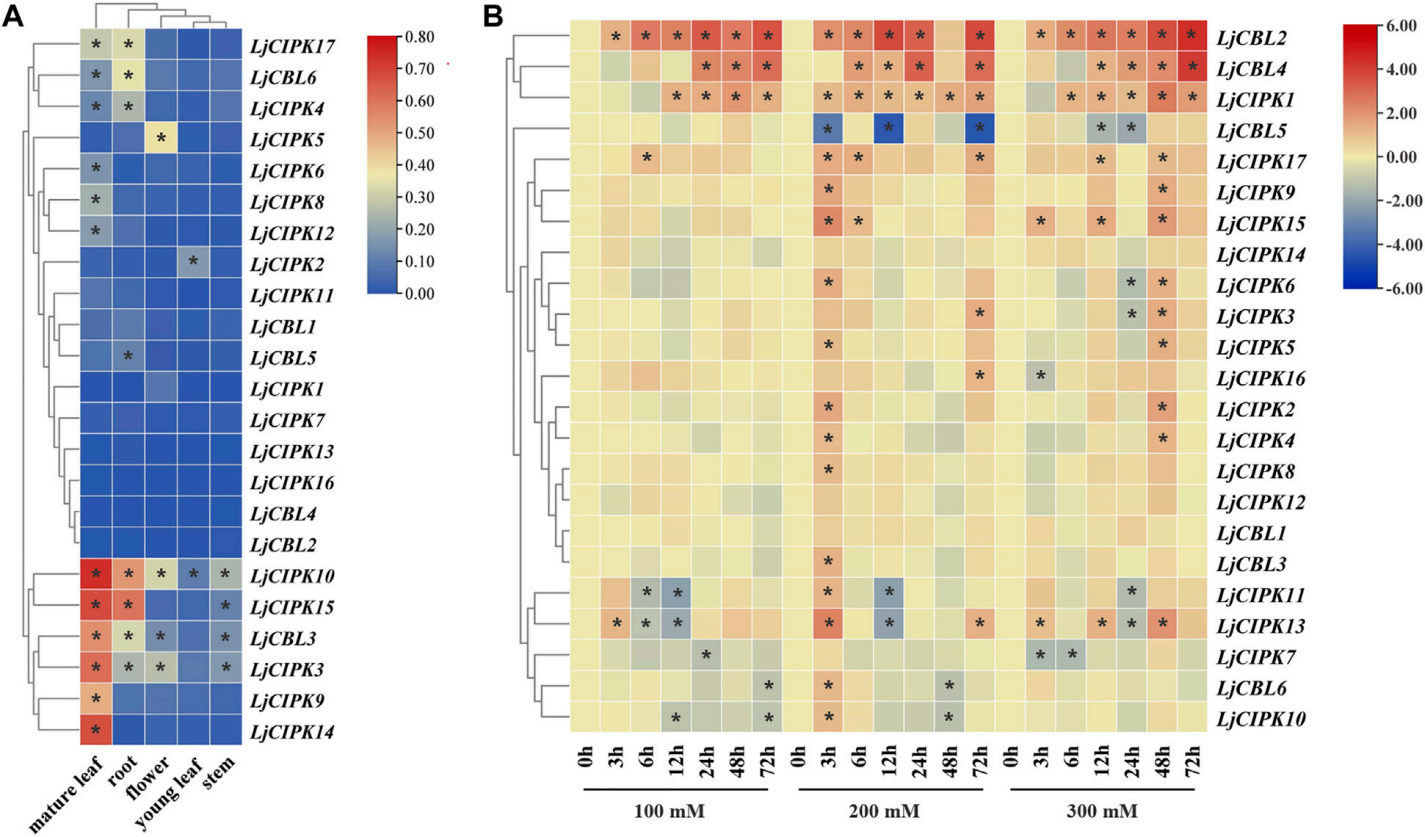
Expression patterns of CBL-CIPK genes in different tissues (A) and under salt stresses (B) **(A)** The relative mRNA abundance of the CBL-CIPK was quantified in five tissues. All values were expressed relative to the expression levels of reference genes using formula 2^−ΔCt^ (Wang et al., 2020). Different colors indicate different levels of gene expression; the asterisk indicates the relative expression level was less or greater than 0.05 **(B)** The expression patterns of the CBL-CIPK at 0, 3, 6, 12, 24, 48, and 72 h after treated with NaCl (100, 200, and 300 mM NaCl). All values were expressed relative to the expression levels of reference genes using formula 2^−ΔΔCt^. Ljactin was used as a marker gene. Different colors indicate different levels of gene expression based on the log2 value of the fold change by RT-qPCR; blue indicates downregulation, and red indicates upregulation. The asterisk indicates significant (*p* < 0.05) up/down-regulated expression (>2-fold).

To investigate the potential roles of LjCBLs and LjCIPKs in different tissues, their expression patterns across five honeysuckle tissues (root, stem, mature leaf, young leaf, flower) were analyzed using qRT-PCR. As illustrated in Figure 6A, the examined genes were expressed in all selected tissue samples, although their expression levels differed among tissues. Hierarchical cluster analysis divides the tissue expression data of CBL into two categories. In the first group, *LjCBL6*, *LjCIPK4*/*17* exhibited a high level of expression in mature leaf and root, while *LjCBL1*/*2*/*4*, *LjCIPK1*/*7*/*11*/*13*/*16* exhibited low levels of expression in almost all tissues. In the second group, *LjCBL3*, *LjCIPK3*/*10* had a high level of expression in almost all tissues. Notably, *LjCBL3* and *LjCIPK3*/*9*/*10*/*14*/*15* showed high specific expression in mature leaves. These results collectively illustrate that LjCBL and LjCIPK genes are important for honeysuckle growth and development, and different genes may have functional variations.

To determine which LjCBL and LjCIPK genes contribute to salt stress tolerance, the time-course change in expression levels of genes was analyzed in the roots of the “Huajin 6” cultivar under different concentrations of NaCl by RT-qPCR. As shown in Figure 6B, the expression of *LjCBL2*/*4*, *LjCIPK1* under all treatments gradually increased over time until peak expression at 72 h. When exposed to moderate (200 mM NaCl) and high salt stresses (300 mM NaCl), the expression of *LjCBL4* was 7.54- and 16.6-fold higher at 72 h than under control conditions, respectively. Notably, *LjCIPK11*/*13* rapidly increased the expression level at 3 h and maintained a lower level from 12 or 24 h, then significantly increased their expression level over a 48-h period.

## Discussion

Honeysuckle plays an irreplaceable role in the development of TCM. The completion of the whole-genome sequencing of honeysuckle (unpublished) makes it possible to analyze various gene families of honeysuckle by bioinformatics. Here, a total of six LjCBL genes and 17 LjCIPK genes have been identified and characterized. There are 10 LjCBL genes and 26 LjCIPK genes in Arabidopsis (*Arabidopsis thaliana*), 10 OsCBL genes and 33 OsCIPK genes in rice (*Oryza sativa*) (Kolukisaoglu et al., 2004; Kanwar et al., 2014), seven VvCBL genes and 20 VvCIPK genes in Grapevine (*Vitis vinifera*) (Xi et al., 2017), 10 PtCBL genes and 27 PtCIPK genes in poplar (*Populus trichocarpa*) (Tang et al., 2010), five SmCBL genes and 15 SmCIPK genes in eggplant (*Solanum melongena*) (Li et al., 2016), eight ZmCBLs and 43 ZmCIPK in maize (*Zea mays*) (Chen et al., 2011), 19 BrrCBL and 51 BrrCIPK genes in Turnip (*Brassica rapa*) (Yin et al., 2017). It is well known that gene duplication and loss events during evolution lead to these differences in the number of CBL-CIPK genes among various species (Tian et al., 2017). The presence of multiple CBL-CIPK genes in the genome of the honeysuckle indicates the functional diversity of the CBL-CIPK gene family.

CBL proteins share an overall structural homology consisting of four elongation factor EF-hand domains responsible for binding Ca^2+^. Differences in the EF-hand motifs may result in different ways of binding to Ca^2+^ (Raymond, 1998; Kim et al., 2000; Tang et al., 2020). As shown in Table 1, EF-hands of LjCBLs were highly conserved in honeysuckle, indicating the functional conservation of binding Ca^2+^ ions. CIPK consists of a conserved N terminal catalytic domain for kinase activity and a less conserved C terminal regulatory region containing the NAF motif/FISL motif for interaction with CBL as well as the protein phosphatase interaction motif for interaction with type-2C protein phosphatases (PP2C) and is separated by a junction domain (Albrecht et al., 2001; Liu et al., 2019). Once signal transduction begins, Ca^2+^-bound CBL interacts with CIPK via the NAF domain and releases the inhibitory effect of the C terminus, leading to activation of the kinase (Guo et al., 2001; Tang et al., 2020). It has been shown that the putative NAF domains are highly conserved in the CIPK gene families, which is specific to mediate the interaction between CBL and CIPK (Zhang et al., 2019). Motif analysis showed that the NAF domain was located in motif 10 of the LjCIPKs and it existed in all the detected CIPKs in honeysuckle. Similar results were found in pepper (*Capsicum annuum*) (Ma X. et al., 2019) and canola (*Brassica napus*) (Zhang et al., 2014). These results indicated the CIPK family genes in honeysuckle are relatively conserved during evolution.

Protein myristoylation and palmitoylation are two critical modifications necessary for protein stability, aggregation, and trafficking (Kim et al., 2000; Hemsley and Grierson, 2008). Myristoylation is an irreversible protein modification in which myristate, a 14-carbon saturated fatty acid, is covalently attached through an amide bond to an N-terminal glycine residue in a co-translational process (Ishitani et al., 2000). In honeysuckle, *LjCBL1*/*2*/*4*/*6* starts from a conserved N-myristoylation site, similar to the CBL protein structures from canola (*Brassica napus*) (Zhang et al., 2014) and rice (*Oryza sativa*) (Gu et al., 2008), which may play a role in the membrane targeting of the CBL-CIPK complex. Palmitoylation is the reversible addition of fatty acids to proteins, which increases their membrane affinity (Hemsley and Grierson, 2008). In Arabidopsis, the S-acylation-dependent (S-acylation, also known as palmitoylation) association of *AtCBL2* with the vacuolar membrane is essential for ABA responses (Hrabak et al., 2003). Consistent with the results of Arabidopsis and canola (*Brassica napus*) (Zhang et al., 2014), all the six LjCBL proteins possess typical palmitoylation sites at the N-terminal.

The phylogenetic analysis serves as an excellent method to analyze evolutionary relationships among genes. Phylogenetic analysis of LjCBL and LjCIPK genes in honeysuckle, together with Arabidopsis and rice, classified both CBLs and CIPKs mainly into four different groups. Similar results have also been found in eggplant (*Solanum melongena*) (Li et al., 2016) and canola (*Brassica napus*) (Zhang et al., 2014). The results also demonstrated that the CBL-CIPK were unevenly distributed among subfamilies, and the CBL-CIPK gene family members in honeysuckle, rice, and Arabidopsis subfamilies. Consistent with the current information of plant evolution, the phylogenetic tree of LjCBLs and LjCIPKs were more closely related to Arabidopsis (dicot) compared to rice (monocot). However, several CBL-CIPK presented in Arabidopsis but was absent in honeysuckle, which mainly because of gene loss. The evolutionary characteristics of exon/intron structure provide strong evidence for phylogenetic grouping (Boudet, 2001). For LjCBLs, all of the CDS was discontinuous by the presence of introns. Whereas, CIPK genes were divided into an exon-poor clade (Group i/ii/iii) and an exon-rich clade (Group iv), similar to those in Arabidopsis (Mao et al., 2016), pepper (Ma X. et al., 2019), and cotton (Cui Y. et al., 2020). Combined with the evolutionary analysis of CIPK in plants, our results indicate that intron gain or loss events were the major driving factors for the gene structural evolution of the CIPK gene family before eudicot-monocot divergence (Zhu et al., 2016; Cui Y. et al., 2020). The structural diversification among the CBL-CIPK genes families may allow CBL-CIPK genes to function differently (Yin et al., 2017).

Gene duplication provides opportunities for the new gene production and its functional divergence in the process of gene family expansion and evolution. The paralogous genes were generated during the divergent evolution from a common ancestral gene through duplication events (segmental or tandem duplication) (Xi et al., 2017). It has been shown that the expansion of the CIPK gene family in *Gossypium hirsutum* and *Gossypium barbadense* mainly due to whole-genome duplication and segmental duplications (Cui Y. et al., 2020). In honeysuckle, only one CIPK paralogous pair (*LjCIPK10*/*LjCIPK15*) was identified and were generated by segmental duplication events, as the genes present on different chromosomes. The Ka/Ks ratio is used to identify whether selective pressure existed on amino acid substitutions (Nekrutenko et al., 2002). Our results suggested that the function of the duplicated CIPK genes in honeysuckle did not diverge much during their evolution course, and purifying selection could mainly contribute to the maintenance of function in CIPK gene families. Noticeably, the duplicated genes of LjCIPKs were only detected in the intron-poor clade, a similar phenomenon has been observed in Grapevine (*Vitis vinifera*), indicating that the intron-poor clade of the LjCIPK gene family may play a more specific role to fulfill the specific characteristics of honeysuckle (Xi et al., 2017).

As a key molecular switch, cis-acting regulatory elements participate in the transcriptional regulation of gene activities that control various biological processes (Wu et al., 2019). Hormones, such as ABA, salicylic acid (SA), auxin (IAA), and gibberellin, play critical roles in several developmental stages and stress response (Gallego-Giraldo et al., 2008; Mishra et al., 2014; Li et al., 2019; Zhang and Li, 2019). Here, seven regulatory elements related to hormones were identified in the promoters of CBL-CIPK genes. ABRE (ABA-responsive element), which belongs to the so-called G-box family, contains an ACGT core, a sequence known to be recognized by plant bZIP proteins. ABRE was found in two-thirds of LjCBL and 13 out of 17 LjCIPK genes, respectively, which was also identified in both Arabidopsis and rice (Gomez-Porras et al., 2007), suggesting that the most CBL-CIPK genes might be involved in the ABA signal pathway, which mainly controls stomatal closure, seed and bud dormancy, and physiological responses to cold, drought, and salinity stress (Mishra et al., 2014). Furthermore, seven stress-responsive regulatory elements were identified namely ARE, W box, LTR, STRE, MBS, TC-rich repeats. Similar elements were found in CBL-CIPKs from Grapevine (*Vitis vinifera*) and BnaNHXs from oilseed rape (*Brassica napus*) (Cui J.-q. et al., 2020). Interestingly, W box (TTGACC) was identified in half of the CBL-CIPK genes from honeysuckle. W box is recognized by the family of WRKY transcription factors, which is involved in certain developmental processes and stress response, such as drought stress response in tomato (Ahammed et al., 2020), salt response in Arabidopsis (Xu et al., 2018) and populus (Jiang et al., 2020). In general, the identified cis-acting elements here help in understanding their roles in the developmental and various biotic stress-related mechanisms.

All CBL-CIPK gene expression displayed tissue specificity, which indicated the distinct effect of CBL-CIPK genes. Interestingly, *LjCBL3* and *LjCIPK3*/*9*/*10*/*14*/*15* were highly expressed in mature leaves. Same result was found in cassava (*Manihot esculenta*), *MeCBL1*/*9* and *MeCIPK23* have higher transcriptional levels in the mature stage (Mo et al., 2018). Since CBL-CIPK is a modulator system for efficient nutrient acquisition, it is speculated that different tissues have different requirements for nutrient ions, resulting in tissue-specific expression of related genes (Dong et al., 2021; Verma et al., 2021). CBL and CIPK genes have been reported to enhance salinity tolerance in different species by establishing the homeostasis of macro-nutrients in the cytosol and subcellular compartments, such as in pigeon pea (*Cajanus cajan*) (Meng et al., 2021) and cotton (*Gossypium hirsutum*) (Sun et al., 2021). Our study revealed that in honeysuckle, several CBL-CIPK genes express differentially at different time intervals under salinity stress. The SOS pathways, consisting of *SOS3*, *SOS2*, and *SOS1*, have been well defined as crucial pathways to control cellular ion homeostasis, by extruding Na^+^ to the extracellular space, thus conferring salt stress resistance in plants. As shown in Table 1, *LjCBL4* exhibited orthologous relationships with *AtSOS3*(At5g24270), while *LjCIPK16* exhibited orthologous relationships with *AtSOS2*(At5g35410). As shown in Figure 1, there is a close evolutionary relationship between *LjCBL4* and *SOS3*, *LjCIPK16* and *SOS2*. Interestingly, *SOS1*, a plasma membrane Na^+^/H^+^ antiporter, was predicted to interact with *LjCIPK15*/*16* and *LjCBL4* in the PPI network, while *NHX1*, the Na^+^/H^+^ antiporter located on tonoplast, was also predicted to interact with *LjCIPK16* and *LjCBL4*, which indicated the key role of *LjCIPK16* and *LjCBL4* proteins in the salt tolerance of honeysuckle. Moreover, *LjCBL4*, *LjCIPK16* and *SOS1* were clustered into green cluster in the PPI network, indicating that they may act in the same pathway. As shown in Figure 6, expression profile analyses revealed that *LjCBL4* and *LjCIPK16* were upregulated under salt stress. The SOS pathway genes upregulated in response to salinity stress were confirmed in many plants, such as spinach (*Spinacia oleracea*) (Zhao et al., 2020) and poplar (*Populus trichocarpa*) (Tang et al., 2010). This result suggested that the Ca^2+^-*SOS3*-*SOS2*-cation/H^+^ antiporters (NHXs/AKTs) pathway might be not only a common but also an essential pathway regulating plant salt stress resistance (Zhao et al., 2020). This result also demonstrated the conservation of SOS pathway genes in honeysuckle and a model of Ca^2+^-*LjCBL4*/*LjSOS3*-*LjCIPK16*/*LjSOS3* module-mediated abiotic stress signaling in honeysuckle is proposed.

At the same time, the functions of *LjCBL2*/*5*, *LjCIPK1*/*11*/*13*/*15*/*17* in the response of honeysuckle to salt stress are worth further exploring. Notably, *LjCIPK1* and *LjCIPK15* show greater enhanced expression under salt stress, even higher than *LjCIPK16*. *LjCIPK15* exhibited orthologous relationships with *AtCIPK6* (At4g30960). In Arabidopsis, overexpression of *AtCIPK6* increased plant tolerance to salt stress (Chen et al., 2013); *cipk6* mutant was more sensitive to salt stress compared to wild-type (Tripathi et al., 2009), which indicated *LjCIPK15* may play significant roles in salt tolerance. *LjCIPK1* exhibited orthologous relationships with *AtCIPK11* (At2g30360). It has been shown that *AtCIPK11* functions as a negative regulator in drought stress response in Arabidopsis, its role in salt tolerance needs deep exploration (Ma Y. et al., 2019). Although the sensitivity of each gene to external environmental changes is different, their role in the response of honeysuckle to salt stress deserves more attention.

## Conclusion

In the present study, a total of six LjCBL and 17 LjCIPK genes were identified. The phylogenetic analysis divided both CBL and CIPK genes into four subgroups and the same clade had similar motif compositions and gene structures. Cis-Acting elements analysis implied that both LjCBLs and LjCIPKs are involved in stress adaptation. PPI network analysis results showed that *LjCBL4* is hypothesized to interact with *LjCIPK7*/*9*/*15*/*16* and *SOS1*/*NHX1*. The salt-induced expression patterns confirmed that the expression levels of *LjCBL2*/*4*, *LjCIPK1*/*15*/*16*/*17* were affected by salinity. The theoretical foundation was established in the present study for the further functional characterization of the CBL-CIPK gene families in honeysuckle. However, extra works are required to decipher the interaction networks between LjCBLs and LjCIPKs, and the regulation mode of CBL-CIPK complexe’s response to salt stress.

## Data Availability

The datasets supporting the conclusions of this article are included within the article and its Supplementary Materials, further inquiries can be directed to the corresponding author, Dr. Jia Li (ljytl7172@163.com).

## Supplementary Material

Supplementary Figure S1 Details of motifs in LjCBL proteins identified by MEME.

Supplementary Figure S2 Details of motifs in LjCIPK proteins identified by MEME.

Supplementary Figure S3 Details of the secondary structure of LjCBL proteins.

Supplementary Figure S4 Details of the secondary structure of LjCIPK proteins.

Supplementary Table S1 Phylogeny sequences. Primer sequence. Pairwise sequence similarity and divergence. The non-synonymous/synonymous (Ka/Ks) ratio. Motif sequences.
