# Supplementary figures and images for: Genome-Wide Identification of CBL-CIPK Gene Family in Honeysuckle (Lonicera japonica Thunb.) and Their Regulated Expression Under Salt Stress
Source: Front Genet. 2021 Nov 2;12:751040. doi: 10.3389/fgene.2021.751040 (PMC8593244; doi:10.3389/fgene.2021.751040)

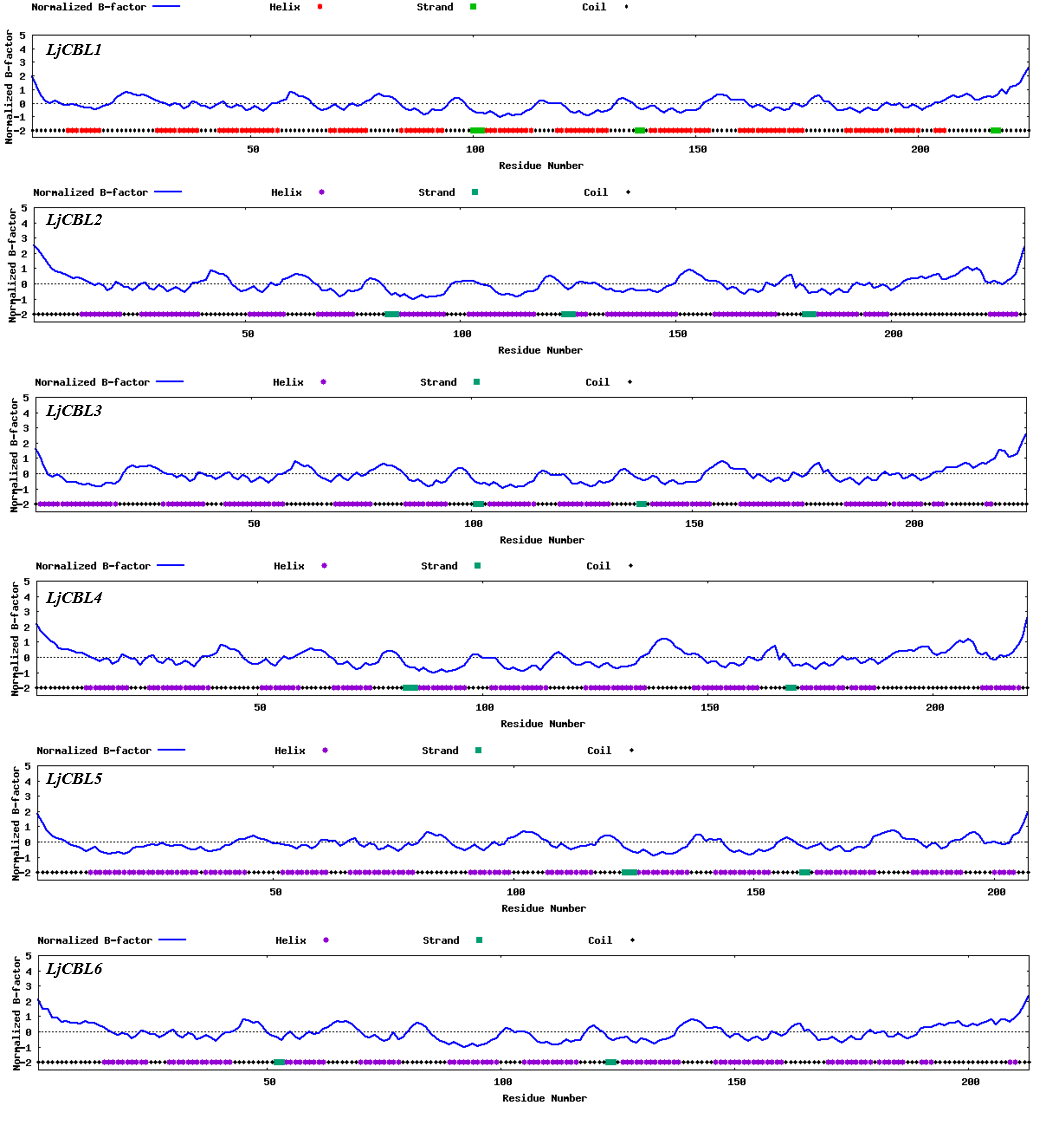

Supplement: Supplementary file 1 [file Image3.TIF]

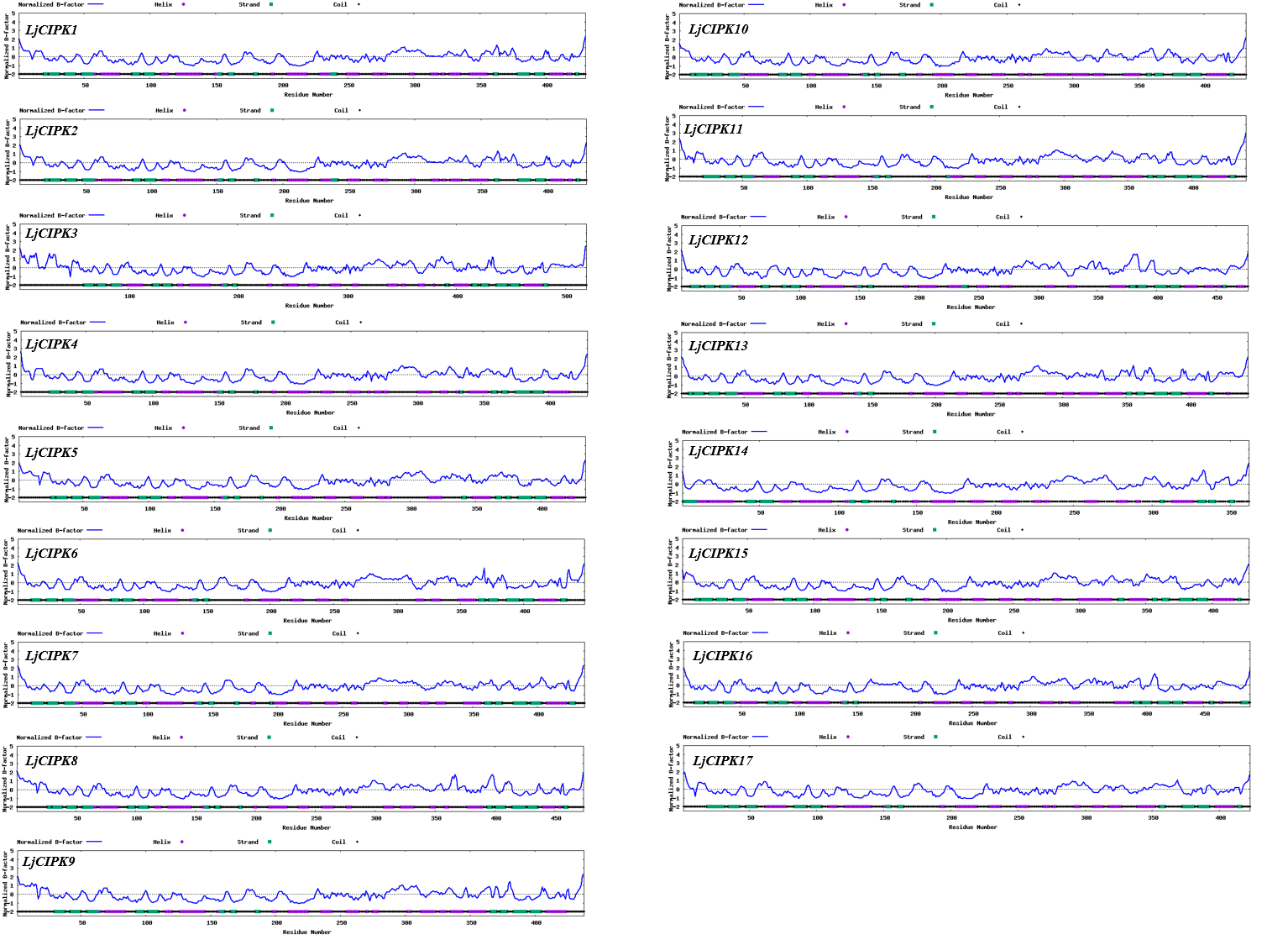

Supplement: Supplementary file 2 [file Image4.TIF]

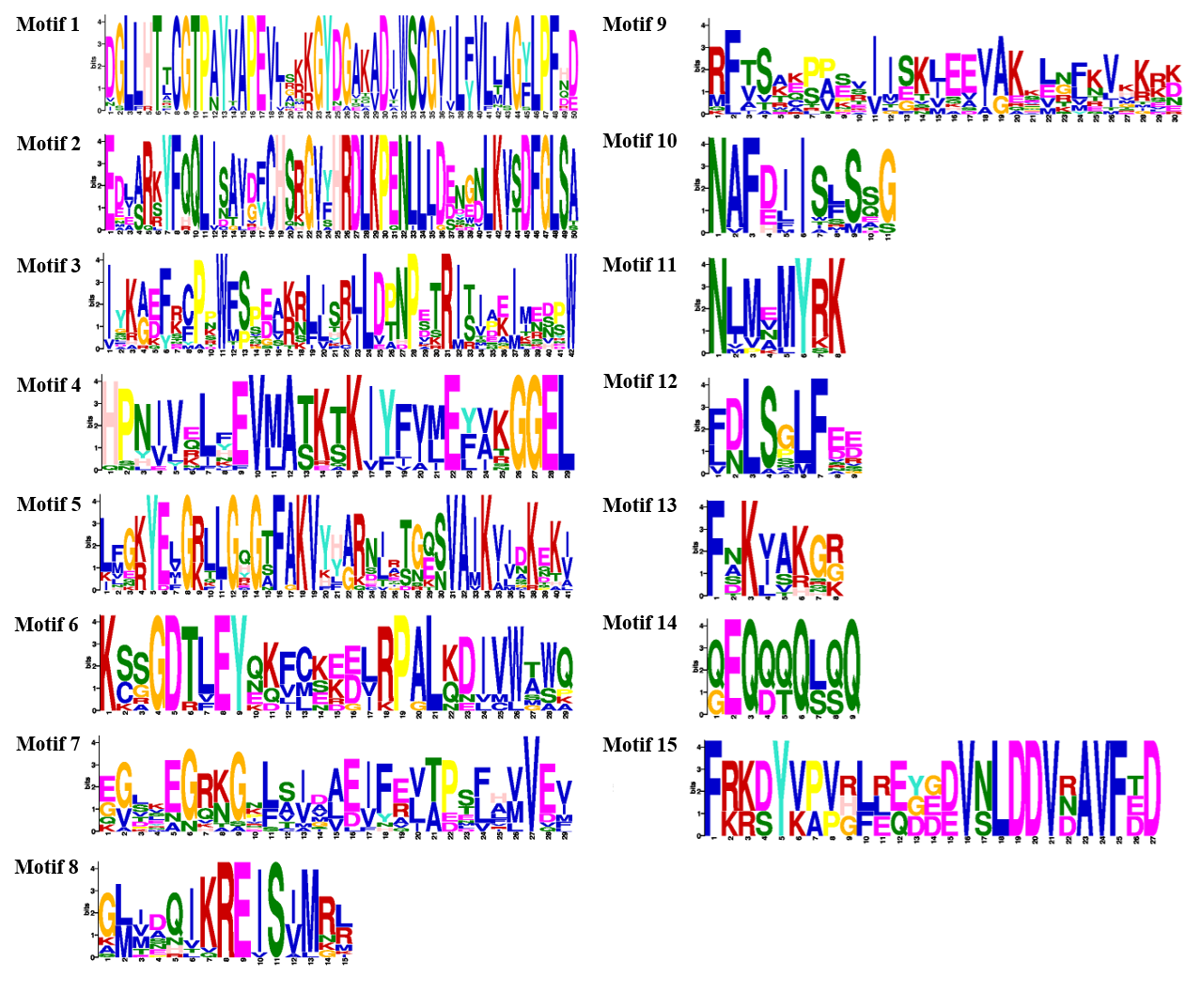

Supplement: Supplementary file 3 [file Image2.TIF]

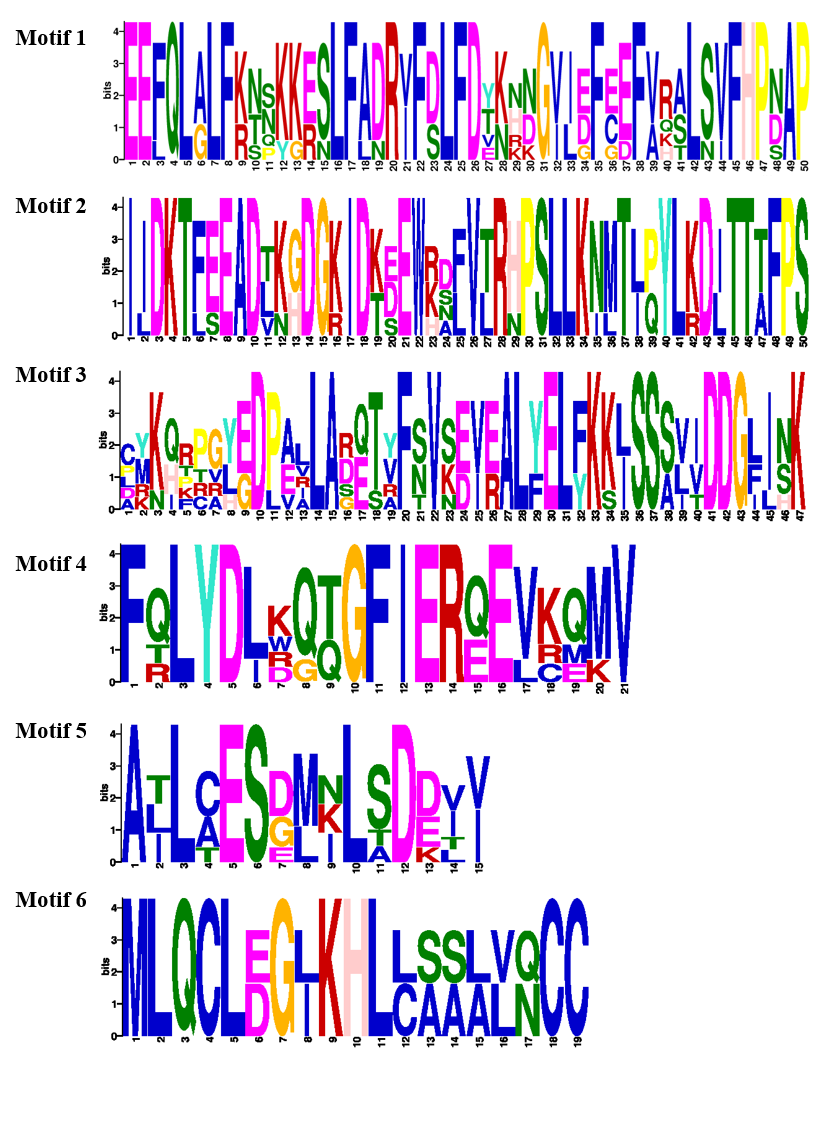

Supplement: Supplementary file 4 [file Image1.TIF]
